# Ethane-1,2-diaminium 4,5-dichloro­phthalate

**DOI:** 10.1107/S1600536809054427

**Published:** 2009-12-24

**Authors:** Graham Smith, Urs D. Wermuth

**Affiliations:** a School of Physical and Chemical Sciences, Queensland University of Technology, GPO Box 2434, Brisbane, Queensland 4001, Australia; b School of Biomolecular and Physical Sciences, Griffith University, Nathan, Queensland 4111, Australia

## Abstract

In the structure of the title compound, C_2_H_10_N_2_^2+^·C_8_H_2_Cl_2_O_4_^2−^, the dications and dianions form hydrogen-bonded ribbon substructures which enclose conjoint cyclic *R*_2_^1^(7), *R*_1_^2^(7) and *R*_4_^2^(8) associations and extend down the *c*-axis direction. These ribbons inter-associate down *b*, giving a two-dimensional sheet structure. In the dianions, one of the carboxyl­ate groups is essentially coplanar with the benzene ring, while the other is normal to it [C—C—C—O torsion angles = 177.67 (12) and 81.94 (17)°, respectively].

## Related literature

For structures of 4,5-dichloro­phthalates, see: Mattes & Dorau (1986 ▶); Bozkurt *et al.* (2006 ▶); Smith & Wermuth (2010*a* ▶,*b* ▶); Smith *et al.* (2009 ▶). For the structure of a dianionic 4,5-dichloro­phthalate, see: Büyükgüngör & Odabaşoğlu (2007 ▶). For ring associations, see: Etter *et al.* (1990 ▶).
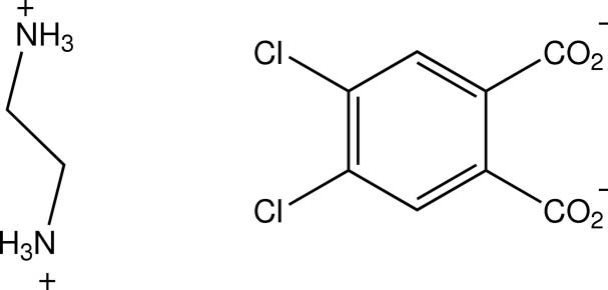


## Experimental

### 

#### Crystal data

C_2_H_10_N_2_^2+^·C_8_H_2_Cl_2_O_4_^2−^
*M* *_r_* = 295.12
Monoclinic, 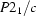

*a* = 12.892 (1) Å
*b* = 8.3881 (5) Å
*c* = 11.8873 (8) Å
β = 104.761 (7)°
*V* = 1243.06 (15) Å^3^
*Z* = 4
Mo *K*α radiation
μ = 0.53 mm^−1^
*T* = 200 K
0.35 × 0.30 × 0.28 mm

#### Data collection

Oxford Diffraction Gemini-S CCD detector diffractometer
Absorption correction: multi-scan (*SADABS*; Sheldrick, 1996 ▶) *T*_min_ = 0.955, *T*_max_ = 0.980
15108 measured reflections
2442 independent reflections
2174 reflections with *I* > 2σ(*I*)
*R*_int_ = 0.025

#### Refinement

*R*[*F* ^2^ > 2σ(*F* ^2^)] = 0.026
*wR*(*F* ^2^) = 0.071
*S* = 1.09
2442 reflections
187 parameters
H atoms treated by a mixture of independent and constrained refinement
Δρ_max_ = 0.28 e Å^−3^
Δρ_min_ = −0.24 e Å^−3^

### 

Data collection: *CrysAlis PRO* (Oxford Diffraction, 2009 ▶); cell refinement: *CrysAlis PRO*; data reduction: *CrysAlis PRO*; program(s) used to solve structure: *SIR92* (Altomare *et al.*, 1994 ▶); program(s) used to refine structure: *SHELXL97* (Sheldrick, 2008 ▶) within *WinGX* (Farrugia, 1999 ▶); molecular graphics: *PLATON* (Spek, 2009 ▶); software used to prepare material for publication: *PLATON*.

## Supplementary Material

Crystal structure: contains datablocks global, I. DOI: 10.1107/S1600536809054427/pv2246sup1.cif

Structure factors: contains datablocks I. DOI: 10.1107/S1600536809054427/pv2246Isup2.hkl

Additional supplementary materials:  crystallographic information; 3D view; checkCIF report

## Figures and Tables

**Table 1:** Hydrogen-bond geometry (Å, °)

*D*—H⋯*A*	*D*—H	H⋯*A*	*D*⋯*A*	*D*—H⋯*A*
N1*A*—H11*A*⋯O21^i^	0.990 (17)	1.756 (17)	2.7452 (16)	177.3 (18)
N1*A*—H12*A*⋯O22^ii^	0.91 (2)	1.88 (2)	2.7709 (16)	168.3 (16)
N1*A*—H13*A*⋯O12^iii^	0.91 (2)	1.90 (2)	2.7876 (17)	163.8 (17)
N2*A*—H21*A*⋯O12^iii^	0.907 (19)	1.974 (18)	2.8306 (16)	157.0 (16)
N2*A*—H21*A*⋯O22^iii^	0.907 (19)	2.502 (19)	3.0370 (17)	118.2 (13)
N2*A*—H22*A*⋯O11^iv^	0.921 (18)	1.824 (19)	2.7221 (17)	164.2 (17)
N2*A*—H23*A*⋯O22	0.922 (18)	1.922 (18)	2.7619 (16)	150.5 (18)

Symmetry codes: (i) 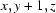; (ii) 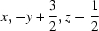; (iii) 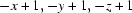; (iv) 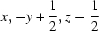.

**Fig. 1.**
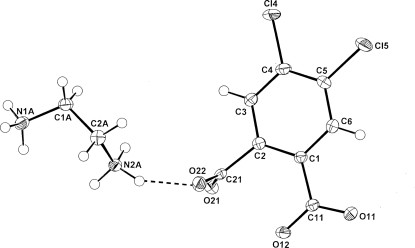
Molecular structure and atom numbering scheme for the ethylenediaminium cation and the 4,5-dichlorophthalate dianion in (I). Non-H atoms are shown as 50% probability displacement ellipsoids. The inter-species hydrogen bond is shown as a dashed line.

**Fig. 2.**
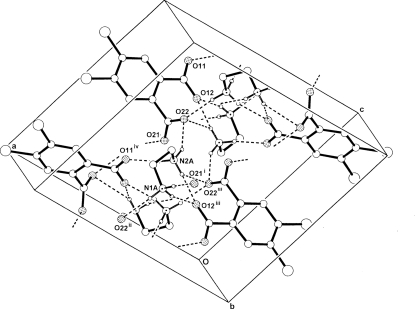
The one-dimensional hydrogen-bonded ribbon substructures of (I) extending along the c axial direction in the unit cell. Hydrogen bonds are shown as dashed lines and non-interactive H atoms are omitted. For symmetry codes see Table 1.

**Fig. 3.**
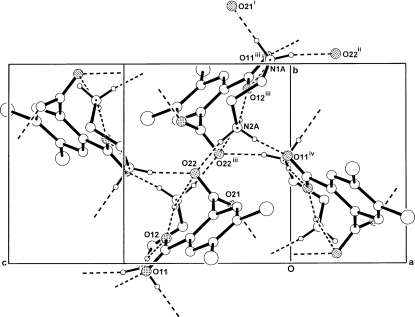
The inter-ribbon hydrogen-bonding in the two-dimensional structure of (I) extending down b axis in the unit cell.

## supplementary crystallographic information

### Comment

The structures of proton-transfer compounds of 4,5-dichlorophthalic acid (DCPA) with the aliphatic nitrogen Lewis bases are not common. The 1:1 ammonium and the tetra(*n*-butyl)ammonium salts (Mattes & Dorau, 1986), the tetramethylammonium salt (Bozkurt *et al.*, 2006) and the salts with the aliphatic amines isopropylamine (Smith & Wermuth, 2010a), diisopropylamine and hexamethylenediamine (a monohydrate) (Smith & Wermuth, 2010b) constitute the only reported examples. With one exception, the ammonium salt (a monohydrate) (Mattes & Dorau, 1986), one-dimensional hydrogen-bonded chain structures are found. In these, the DCPA anions are essentially planar with a short intramolecular carboxylic acid *O*–*H*···O_carboxyl_ hydrogen bond. These 'planar' anions are also found in the majority of the hydrogen DCPA salts of the aromatic Lewis bases (Smith *et al.*,  2009). The structures of dianionic 4,5-dichlorophthalate salts with any Lewis base are also uncommon, the only known example being the bis(4-ethylanilinium) salt (Büyükgüngör & Odabaşoğlu, 2007). As expected in the structure of the salt reported here, C_2_H_10_N_2_^2+^ C_8_H_3_Cl_2_O_4_^2-^ (I), the DCPA dianions are nonplanar.

The title compound (I) was the product obtained from the 1:1 stoichiometric reaction of DCPA with ethylenediamine in methanol but suitable crystals were obtained only after recrystallization from water. In (I) (Fig. 1), the ethylenediaminium dications and the DCPA dianions form head-to-tail *N*^+^–*H*···O_carboxyl_ hydrogen-bonding interactions (Table 1), forming crosslinked duplex chains which extend along the *c* cell direction (Fig. 2). These ribbon structures enclose conjoint *R*_2_^1^(7), *R*_1_^2^(7) and *R*_4_^2^(8) associations (Etter *et al.*, 1990). These ribbons associate down the *b* axial direction, giving a two-dimensional network structure (Fig. 3).

Within the DCPA dianions one of the carboxylate groups is essentially coplanar with the benzene ring [torsion angle C2–C1–C11–O11, 177.67 (12)°] while the other is normal to the plane [torsion angle C1–C2–C21–O22, 81.94 (17)°]. The coplanar carboxyl group is stabilized by a short intramolecular aromatic ring *C*–*H*···O_carboxyl_ interaction [2.7594 (18) Å].

### Experimental

The title compound (I) was synthesized by heating together 1 mmol quantities of ethylenediamine and 4,5-dichlorophthalic acid in 50 ml of methanol for 10 min under reflux. After concentration to *ca*. 30 ml, total room-temperature evaporation of the hot-filtered solution gave a white non-crystalline solid which was redissolved in water, finally providing colourless flat prisms (m.p. 529 K).

### Refinement

Hydrogen atoms of the aminium groups were located from a difference Fourier map and their positional and isotropic displacement parameters were refined. Other H atoms were included in the refinement at calculated positions [C–H_aromatic_ = 0.93 Å; C–H_aliphatic_ = 0.97 Å] and treated as riding models with *U*_iso_(H) = 1.2*U*_eq_ (C).

### Crystal data

**Table:**

C_2_H_10_N_2_^2+^·C_8_H_2_Cl_2_O_4_^2^^−^	*F*(000) = 608
*M**_r_* = 295.12	*D*_x_ = 1.577 Mg m^−^^3^
Monoclinic, *P*2_1_/*c*	Melting point: 529 K
Hall symbol: -P 2yc	Mo *K*α radiation, λ = 0.71073 Å
*a* = 12.892 (1) Å	Cell parameters from 7149 reflections
*b* = 8.3881 (5) Å	θ = 3.2–28.8°
*c* = 11.8873 (8) Å	µ = 0.53 mm^−^^1^
β = 104.761 (7)°	*T* = 200 K
*V* = 1243.06 (15) Å^3^	Block, colourless
*Z* = 4	0.35 × 0.30 × 0.28 mm

### Data collection

**Table:**

Oxford Diffraction Gemini-S CCD detector diffractometer	2442 independent reflections
Radiation source: Enhance (Mo) X-ray source	2174 reflections with *I* > 2σ(*I*)
graphite	*R*_int_ = 0.025
ω scans	θ_max_ = 26.0°, θ_min_ = 3.2°
Absorption correction: multi-scan (*SADABS*; Sheldrick, 1996)	*h* = −15→15
*T*_min_ = 0.955, *T*_max_ = 0.980	*k* = −10→10
15108 measured reflections	*l* = −14→14

### Refinement

**Table:**

Refinement on *F*^2^	Primary atom site location: structure-invariant direct methods
Least-squares matrix: full	Secondary atom site location: difference Fourier map
*R*[*F*^2^ > 2σ(*F*^2^)] = 0.026	Hydrogen site location: inferred from neighbouring sites
*wR*(*F*^2^) = 0.071	H atoms treated by a mixture of independent and constrained refinement
*S* = 1.09	*w* = 1/[σ^2^(*F*_o_^2^) + (0.0401*P*)^2^ + 0.2917*P*] where *P* = (*F*_o_^2^ + 2*F*_c_^2^)/3
2442 reflections	(Δ/σ)_max_ = 0.001
187 parameters	Δρ_max_ = 0.28 e Å^−^^3^
0 restraints	Δρ_min_ = −0.24 e Å^−^^3^

### Special details

**Table:**

Geometry. Bond distances, angles *etc*. have been calculated using the rounded fractional coordinates. All su's are estimated from the variances of the (full) variance-covariance matrix. The cell e.s.d.'s are taken into account in the estimation of distances, angles and torsion angles
Refinement. Refinement of *F*^2^ against ALL reflections. The weighted *R*-factor *wR* and goodness of fit *S* are based on *F*^2^, conventional *R*-factors *R* are based on *F*, with *F* set to zero for negative *F*^2^. The threshold expression of *F*^2^ > σ(*F*^2^) is used only for calculating *R*-factors(gt) *etc*. and is not relevant to the choice of reflections for refinement. *R*-factors based on *F*^2^ are statistically about twice as large as those based on *F*, and *R*- factors based on ALL data will be even larger.

### Fractional atomic coordinates and isotropic or equivalent isotropic displacement parameters (Å^2^)

**Table:**

	*x*	*y*	*z*	*U*_iso_*/*U*_eq_	
Cl4	1.04287 (3)	0.27191 (5)	0.51216 (3)	0.0296 (1)	
Cl5	1.08544 (3)	0.02709 (6)	0.72577 (4)	0.0387 (2)	
O11	0.70606 (8)	−0.03906 (13)	0.79920 (9)	0.0278 (3)	
O12	0.59071 (7)	0.12464 (13)	0.68133 (9)	0.0241 (3)	
O21	0.58818 (8)	0.28845 (12)	0.44570 (8)	0.0219 (3)	
O22	0.63839 (8)	0.45072 (12)	0.59914 (8)	0.0212 (3)	
C1	0.77314 (10)	0.12670 (16)	0.67229 (11)	0.0161 (4)	
C2	0.75625 (11)	0.24125 (16)	0.58269 (11)	0.0157 (3)	
C3	0.83974 (11)	0.28193 (16)	0.53312 (12)	0.0179 (4)	
C4	0.94056 (11)	0.21453 (17)	0.57457 (12)	0.0199 (4)	
C5	0.95892 (11)	0.10573 (18)	0.66647 (13)	0.0218 (4)	
C6	0.87486 (11)	0.06009 (17)	0.71298 (12)	0.0209 (4)	
C11	0.68289 (10)	0.06677 (16)	0.72250 (11)	0.0164 (4)	
C21	0.65154 (11)	0.33073 (16)	0.53918 (11)	0.0160 (4)	
N1A	0.60404 (10)	1.02002 (15)	0.31902 (11)	0.0207 (4)	
N2A	0.58594 (10)	0.68181 (14)	0.42855 (11)	0.0180 (3)	
C1A	0.69095 (11)	0.91156 (17)	0.38430 (13)	0.0209 (4)	
C2A	0.65833 (11)	0.81624 (17)	0.47846 (12)	0.0212 (4)	
H3	0.82780	0.35450	0.47200	0.0220*	
H6	0.88660	−0.01580	0.77190	0.0250*	
H11A	0.6007 (14)	1.117 (2)	0.3654 (15)	0.034 (5)*	
H12A	0.6168 (15)	1.044 (2)	0.2495 (18)	0.040 (5)*	
H13A	0.5379 (16)	0.975 (2)	0.3039 (16)	0.033 (5)*	
H14A	0.75380	0.97470	0.41970	0.0250*	
H15A	0.71040	0.83840	0.32990	0.0250*	
H21A	0.5195 (15)	0.718 (2)	0.3925 (15)	0.029 (5)*	
H22A	0.6153 (15)	0.623 (2)	0.3789 (16)	0.036 (5)*	
H23A	0.5786 (15)	0.610 (2)	0.4847 (17)	0.038 (5)*	
H24A	0.72210	0.77460	0.53250	0.0250*	
H25A	0.62220	0.88600	0.52140	0.0250*	

### Atomic displacement parameters (Å^2^)

**Table:**

	*U*^11^	*U*^22^	*U*^33^	*U*^12^	*U*^13^	*U*^23^
Cl4	0.0202 (2)	0.0375 (2)	0.0362 (2)	−0.0003 (2)	0.0163 (2)	0.0087 (2)
Cl5	0.0177 (2)	0.0531 (3)	0.0476 (3)	0.0126 (2)	0.0126 (2)	0.0243 (2)
O11	0.0252 (5)	0.0276 (6)	0.0354 (6)	0.0057 (4)	0.0163 (5)	0.0159 (5)
O12	0.0151 (5)	0.0308 (6)	0.0275 (5)	0.0017 (4)	0.0073 (4)	0.0096 (4)
O21	0.0203 (5)	0.0256 (6)	0.0190 (5)	0.0050 (4)	0.0038 (4)	−0.0004 (4)
O22	0.0263 (5)	0.0179 (5)	0.0222 (5)	0.0040 (4)	0.0113 (4)	0.0004 (4)
C1	0.0164 (6)	0.0151 (6)	0.0183 (7)	−0.0008 (5)	0.0070 (5)	−0.0009 (5)
C2	0.0169 (6)	0.0146 (6)	0.0162 (6)	−0.0008 (5)	0.0054 (5)	−0.0021 (5)
C3	0.0199 (7)	0.0177 (7)	0.0173 (7)	−0.0013 (5)	0.0067 (5)	0.0017 (5)
C4	0.0178 (7)	0.0212 (7)	0.0235 (7)	−0.0028 (5)	0.0106 (6)	−0.0009 (6)
C5	0.0151 (7)	0.0243 (8)	0.0267 (7)	0.0040 (6)	0.0067 (6)	0.0035 (6)
C6	0.0202 (7)	0.0211 (7)	0.0220 (7)	0.0021 (6)	0.0067 (6)	0.0066 (6)
C11	0.0178 (7)	0.0149 (6)	0.0180 (7)	−0.0013 (5)	0.0073 (5)	−0.0012 (5)
C21	0.0180 (7)	0.0156 (7)	0.0172 (7)	0.0008 (5)	0.0098 (5)	0.0040 (5)
N1A	0.0227 (7)	0.0195 (6)	0.0224 (6)	−0.0010 (5)	0.0105 (5)	0.0022 (5)
N2A	0.0183 (6)	0.0159 (6)	0.0220 (6)	0.0010 (5)	0.0092 (5)	0.0003 (5)
C1A	0.0181 (7)	0.0183 (7)	0.0283 (7)	−0.0013 (5)	0.0095 (6)	−0.0008 (6)
C2A	0.0220 (7)	0.0207 (7)	0.0202 (7)	−0.0012 (6)	0.0041 (6)	−0.0020 (6)

### Geometric parameters (Å, °)

**Table:**

Cl4—C4	1.7379 (15)	C1—C11	1.5220 (19)
Cl5—C5	1.7340 (15)	C1—C6	1.394 (2)
O11—C11	1.2529 (17)	C2—C21	1.516 (2)
O12—C11	1.2614 (16)	C2—C3	1.395 (2)
O21—C21	1.2511 (16)	C3—C4	1.388 (2)
O22—C21	1.2694 (17)	C4—C5	1.397 (2)
N1A—C1A	1.497 (2)	C5—C6	1.390 (2)
N2A—C2A	1.4874 (19)	C3—H3	0.9300
N1A—H13A	0.91 (2)	C6—H6	0.9300
N1A—H12A	0.91 (2)	C1A—C2A	1.520 (2)
N1A—H11A	0.990 (17)	C1A—H14A	0.9700
N2A—H22A	0.921 (18)	C1A—H15A	0.9700
N2A—H21A	0.907 (19)	C2A—H24A	0.9700
N2A—H23A	0.922 (18)	C2A—H25A	0.9700
C1—C2	1.4096 (18)		
			
H12A—N1A—H13A	107.0 (17)	C4—C5—C6	119.81 (13)
H11A—N1A—H13A	106.3 (16)	C1—C6—C5	120.77 (13)
C1A—N1A—H12A	108.8 (12)	O11—C11—C1	117.07 (12)
C1A—N1A—H13A	113.0 (11)	O12—C11—C1	117.36 (11)
C1A—N1A—H11A	110.1 (10)	O11—C11—O12	125.55 (13)
H11A—N1A—H12A	111.7 (15)	O21—C21—O22	124.97 (13)
C2A—N2A—H22A	110.1 (12)	O21—C21—C2	119.07 (12)
C2A—N2A—H23A	112.1 (12)	O22—C21—C2	115.84 (11)
C2A—N2A—H21A	110.8 (11)	C2—C3—H3	120.00
H21A—N2A—H23A	107.6 (17)	C4—C3—H3	120.00
H22A—N2A—H23A	104.4 (16)	C1—C6—H6	120.00
H21A—N2A—H22A	111.7 (16)	C5—C6—H6	120.00
C2—C1—C6	119.08 (13)	N1A—C1A—C2A	112.98 (12)
C2—C1—C11	122.35 (12)	N2A—C2A—C1A	111.62 (11)
C6—C1—C11	118.50 (12)	N1A—C1A—H14A	109.00
C3—C2—C12	116.79 (12)	N1A—C1A—H15A	109.00
C1—C2—C3	119.92 (13)	C2A—C1A—H14A	109.00
C1—C2—C12	123.24 (12)	C2A—C1A—H15A	109.00
C2—C3—C4	120.28 (13)	H14A—C1A—H15A	108.00
C3—C4—C5	120.05 (13)	N2A—C2A—H24A	109.00
Cl4—C4—C5	121.27 (11)	N2A—C2A—H25A	109.00
Cl4—C4—C3	118.67 (11)	C1A—C2A—H24A	109.00
Cl5—C5—C4	121.40 (11)	C1A—C2A—H25A	109.00
Cl5—C5—C6	118.78 (11)	H24A—C2A—H25A	108.00
			
C6—C1—C2—C3	2.4 (2)	C1—C2—C21—O22	81.94 (17)
C6—C1—C2—C12	−174.79 (12)	C3—C2—C21—O21	80.75 (17)
C11—C1—C2—C3	−174.55 (12)	C3—C2—C21—O22	−95.33 (15)
C11—C1—C2—C12	8.3 (2)	C2—C3—C4—Cl4	−178.67 (11)
C2—C1—C6—C5	0.2 (2)	C2—C3—C4—C5	−0.2 (2)
C11—C1—C6—C5	177.24 (13)	Cl4—C4—C5—Cl5	1.97 (18)
C2—C1—C11—O11	177.67 (12)	Cl4—C4—C5—C6	−178.80 (11)
C2—C1—C11—O12	−0.51 (19)	C3—C4—C5—Cl5	−176.52 (11)
C6—C1—C11—O11	0.70 (18)	C3—C4—C5—C6	2.7 (2)
C6—C1—C11—O12	−177.48 (12)	Cl5—C5—C6—C1	176.53 (11)
C1—C2—C3—C4	−2.4 (2)	C4—C5—C6—C1	−2.7 (2)
C12—C2—C3—C4	174.95 (12)	N1A—C1A—C2A—N2A	76.45 (15)
C1—C2—C21—O21	−101.99 (16)		

### Hydrogen-bond geometry (Å, °)

**Table:**

*D*—H···*A*	*D*—H	H···*A*	*D*···*A*	*D*—H···*A*
N1A—H11A···O21^i^	0.990 (17)	1.756 (17)	2.7452 (16)	177.3 (18)
N1A—H12A···O22^ii^	0.91 (2)	1.88 (2)	2.7709 (16)	168.3 (16)
N1A—H13A···O12^iii^	0.91 (2)	1.90 (2)	2.7876 (17)	163.8 (17)
N2A—H21A···O12^iii^	0.907 (19)	1.974 (18)	2.8306 (16)	157.0 (16)
N2A—H21A···O22^iii^	0.907 (19)	2.502 (19)	3.0370 (17)	118.2 (13)
N2A—H22A···O11^iv^	0.921 (18)	1.824 (19)	2.7221 (17)	164.2 (17)
N2A—H23A···O22	0.922 (18)	1.922 (18)	2.7619 (16)	150.5 (18)
C6—H6···O11	0.93	2.44	2.7594 (18)	100
C1A—H15A···O11^iv^	0.97	2.54	3.3052 (18)	136

Symmetry codes: (i) *x*, *y*+1, *z*; (ii) *x*, −*y*+3/2, *z*−1/2; (iii) −*x*+1, −*y*+1, −*z*+1; (iv) *x*, −*y*+1/2, *z*−1/2.
